# Intermittent feeding and circadian rhythm in critical illness

**DOI:** 10.1097/MCC.0000000000000960

**Published:** 2022-07-05

**Authors:** Imre W.K. Kouw, Leonie K. Heilbronn, Arthur R.H. van Zanten

**Affiliations:** a Division of Human Nutrition and Health, Wageningen University & Research, Wageningen, The Netherlands; b Intensive Care Unit, Royal Adelaide Hospital, Adelaide, South Australia, Australia; c Adelaide Medical School, The University of Adelaide, Adelaide, South Australia, Australia; d Centre of Research Excellence in Translating Nutritional Science to Good Health, The University of Adelaide, Adelaide, South Australia, Australia; e Lifelong Health Theme, South Australian Health and Medical Research Institute, Adelaide, South Australia, Australia; f Department of Intensive Care Medicine, Gelderse Vallei Hospital, Ede, The Netherlands

**Keywords:** circadian rhythm, enteral nutrition, metabolic outcomes, timing

## Abstract

Circadian rhythms, i.e., periodic oscillations in internal biological processes, modulate metabolic processes such as hormonal signalling, nutrient absorption, and xenobiotic detoxification. Meal timing is a strong entraining cue for peripheral clocks in various organs, and eating out of circadian phases can impair glucose, gastrointestinal, and muscle metabolism. Sleep/wake cycles and circadian rhythms are extremely disrupted during critical illness. Timing of nutritional support may help preserve circadian rhythms and improve post-Intensive Care Unit (ICU) recovery. This review summarises circadian disruptors during ICU admission and evaluates the potential benefits of intermittent feeding on metabolism and circadian rhythms.

**Recent findings:**

Rhythmic expression of core clock genes becomes rapidly disturbed during critical illness and remains disturbed for weeks. Intermittent, bolus, and cyclic enteral feeding have been directly compared to routine continuous feeding, yet no benefits on glycaemic control, gastrointestinal tolerance, and muscle mass have been observed and impacts of circadian clocks remain untested.

**Summary:**

Aligning timing of nutritional intake, physical activity, and/or medication with circadian rhythms are potential strategies to reset peripheral circadian rhythms and may enhance ICU recovery but is not proven beneficial yet. Therefore, selecting intermittent feeding over continuous feeding must be balanced against the pros and cons of clinical practice.

## INTRODUCTION

Although the survival rates in critically ill patients are increasing worldwide, longer-term outcomes after admission in the Intensive Care Unit (ICU) are often poor, with up to 80% of patients suffering from long-term complications including impairments in sleep, physical function, and cognitive and psychological health [1]. Circadian rhythms, i.e., 24-h cycles, are central to physiological, psychological, and behavioural processes. Disruptions in circadian rhythms are associated with complications such as immune system disruption, delirium, long-term cardiovascular consequences, neurodegenerative diseases, type 2 diabetes mellitus, and increased mortality [2^▪^,3^▪^]. With the ICU environment being so drastically different from daily life with ongoing clinical and environmental changes, these disruptors likely contribute to impairments in circadian rhythms. Supporting circadian health in critically ill patients may help improve metabolism and reduce psychological health impairment and delirium during the post-ICU recovery phase. Therefore, it is essential to understand how critical illness affects circadian rhythms in order to develop intervention strategies and chronotherapy to minimise disruption of patients’ circadian rhythms in the ICU.

Nutritional support forms an essential part of standard clinical care in critically ill patients, thereby improving clinical outcomes. Although current nutritional guidelines [4,5] specify recommendations on the *quantity* and *quality* of the provided energy, macro- and micronutrients, strategies towards the timing and mode of feeding have been largely understudied. *When* food is consumed affects various physiological functions, including the sleep/wake cycle, core body temperature, (skeletal muscle) insulin sensitivity, whole-body metabolic health, and mental alertness. This has been referred to ‘chrononutrition’, i.e. synchronisation of eating with the body's entrained circadian rhythms, which has led to an enormous scientific and public interest in time-restricted eating diets; a dietary strategy that alters meal timing and incorporates more extended daily periods of fasting into the diet, without restricting the total energy intake. Time-restricted eating has been shown to reduce risk factors for type 2 diabetes mellitus and cardiovascular disease [2^▪^,6^▪^].

In the ICU, continuous and intermittent feeding (intermittent, bolus, or cyclic) are the most common enteral nutrition administration strategies. Continuous feeding is standard practice, as the slow release of nutrients into the stomach is thought to enhance feeding tolerance, reduce the risk of regurgitation, and lower respiratory complications, as well as being convenient. In contrast, intermittent feeding is more physiological as it mimics eating patterns in everyday life, thereby maintaining regular gastrointestinal hormone secretion and digestion, and it gives patients more mobility. Studies in animals and healthy humans [7–11] have suggested that intermittent feeding results in improved insulin sensitivity, increased muscle protein synthesis, activation of fasting-induced autophagy and ketogenesis, and the preservation of circadian rhythms in contrast to continuous feeding. However, in critically ill patients, only a handful of studies have directly compared the effect of intermittent versus continuous feeding on clinical outcomes, and these have been discussed in earlier reviews [12–17]. This review aims to provide an overview of studies published in the last 18 months on the effect of timing of nutritional support on metabolic outcomes in critically ill patients, with a specific interest in circadian alignment during and post-ICU admission. 

**Box 1:**
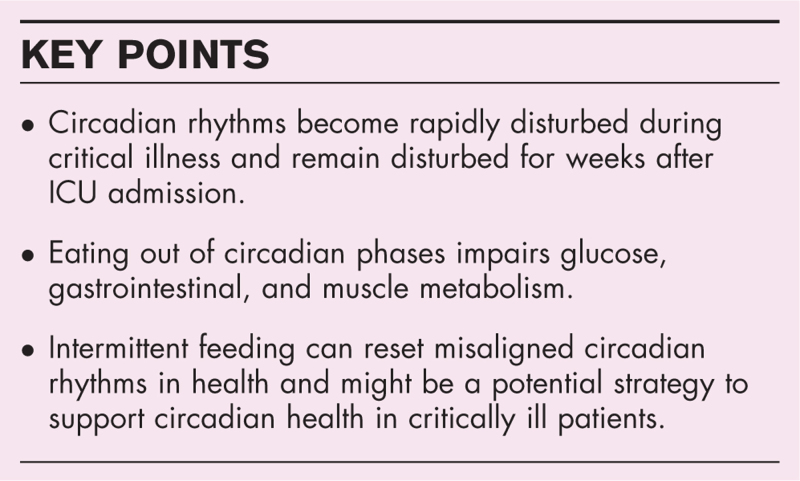
no caption available

## REGULATION OF CIRCADIAN RHYTHMS

Multiple physiological processes in peripheral tissues such as gastrointestinal function, muscle, and other vital organs are all under circadian regulation. The master regulator is in the hypothalamus's suprachiasmatic nucleus, primarily entrained by the light/dark cycle. At the molecular level, the circadian clock is based on the transcriptional/translational feedback loop of proteins such as Cryptochrome (CRY), Period (PER), Brain and muscle Arnt-like protein (BMAL), and Circadian Locomotor Output Cycles Kaput (CLOCK) that take ∼24 h to complete. However, nutrient signalling molecules directly regulate clock genes; activation of insulin-mTOR pathways increases the stability and translation of PER proteins [18,19,20^▪▪^], whereas fasting activates AMP-activated protein kinase (AMPK) and nicotinamide phosphoribosyltransferase (NAMPT) pathways reducing the stability and transcription of CRY and PER [21–23]. In this way, changes in insulin and cAMP due to mistimed meals will influence hundreds of downstream ‘clock-controlled’ genes in peripheral tissues responsible for metabolic processes, including gastrointestinal function, glycaemic control, and muscle metabolism.

Environmental cues such as light/dark phase, temperature changes, and physical activity can synchronise the circadian clock with the external environment, with food consumption being the most potent entrainer for peripheral clocks. In the gut, nutrient uptake, gastric motility, gastric acid and gastrointestinal hormones production, nutrient absorption, and the gut microbiome are under circadian regulation [24^▪▪^,^25^^▪▪^]. Glucose metabolism is also under circadian control [26]; the hepatic clocks regulate glucose production, whereas the pancreatic clocks regulate insulin secretion according to time of day with much less secretory capacity at night. In contrast, the muscle clock regulates glucose uptake through reduced glucose transporter translocation at night versus day [27^▪^]. Moreover, circadian disruption can acutely impact glycaemic control through impairments in beta-cell function and peripheral insulin sensitivity [26].

## THE INTENSIVE CARE UNIT ENVIRONMENT AND CIRCADIAN DISRUPTORS

Diurnal rhythms of central phase markers such as body temperature, blood pressure, heart rate, and sleep patterns in critically ill patients are highly disturbed during ICU admission [28–30] and continue to be disrupted for weeks after discharge [29,31^▪▪^,^32^]. The pathophysiological response to critical illness might primarily drive this disruption in circadian rhythms, whereas non-physiological clinical factors, such as mechanical ventilation, medications, and sedation, may further contribute. Moreover, critical illness comes with pain, fatigue, stress, and cognitive dysfunctions such as delirium, which might further exacerbate circadian disruption [3^▪^]. ICU patients are exposed to frequent patient care interactions, noise, persistent light, and often to continuous enteral feeding, which are potentially modifiable factors that could mitigate circadian disruption. Therefore, further understanding of the extent of circadian disruptors and circadian health in ICU patients is needed.

A handful of studies have assessed the rhythmic expression of clock genes in critically ill patients [28,33–35], and the studies published in the last 18 months are summarised in Table 1. Studies that have quantified circadian rhythms of clock genes in critically ill patients early in ICU admission show no rhythmic expression of crucial clock genes compared to healthy controls [28,33]. However, these studies vary in patient population and are limited to neurology patients and patients with and without sepsis (type of patients not specified). Following admission, the time to inclusion is critical when circadian health is assessed, as circadian rhythm disturbance occurs rapidly [34]. In addition, baseline circadian health (i.e., at home or on the ward) might be variable and is an important confounding factor. For the assessment of circadian disruption (i.e. rhythmicity), the frequency of blood samples is critical for modelling analysis and varied in the published studies from 2- to 6 hourly in 24 h, with recommendations being 2-hourly with at least 4-hourly [36] so as not to underpower the analysis.

**Table 1:** Studies that have assessed circadian disruption in ICU patients in the last 18 months.

Study	Design	Patient population	Methodology	Main findings
Maas *et al.*[33]	Cross-sectional observational study Enrolment within 24 h of emergency department presentation Healthy volunteers were studied in a clinical research facility under similar circumstances	*n* = 15 ICU patients (10 with sepsis and 5 with intracerebral haemorrhage) vs *n* = 11 healthy volunteers	**Primary outcome:** mRNA expression of Cry1–2, Per1–3, RORα, NR1D1, Bmal1, CLOCK, and TIMELESS. Secondary outcomes: Melatonin concentrations (amplitude) Sample analysis: 2-hourly blood samples over 24h Rhythm analysis: Individual consinor fits for each gene along with a population-mean cosinor fit and TimeSignature (a validated algorithm based on 41 genes to evaluate the overall phase coherence of the rhythmic transcriptome)	No rhythmic expression was observed in any clock genes in ICU patients, while circadian rhythmicity was observed in healthy controls (significant cosinor rhythm fit in BMAL1, TIMELESS, CRY1, NR1D1, and PER1).
Diaz *et al.*[34]	Prospective observational study on the first day after ICU admission and 1 week later.	*n* = 11 neuro-ICU patients (*n* = 7 subarachnoid or intracerebral haemorrhage and *n* = 4 traumatic brain injury)	**Primary outcome:** mRNA expression of CLOCK, Bmal1, Cry1, and Per2. Sample analysis: 6-hourly blood samples over 24h (6, 12, 18, 24h after admission) Rhythm analysis: Fourier series and curve fitting	Rhythmicity was observed in all clock genes on the first day after ICU admission, while rhythmicity completely disappeared after one week.
Acuña-Fernández *et al.*[28]	Prospective observational study (*time frame unknown*)	*n* = 24 non-septic ICU patients, *n* = 20 septic ICU patients, and *n* *=* 12 healthy controls	**Primary outcome:** mRNA expression of CLOCK, Bmal1, Cry1 and Per2 Secondary outcomes: Urinary excretion of 6-SM (6-sulfatoxymelatonin) and procalcitonin levels. Sample analysis: 4 blood samples over 24h (at 08:00, 13:00, 18:00, and 23:00h). Rhythm analysis: Relative changes in gene expression.	No difference was detected in Bmal1 and CLOCK expression, while Per2 and Cry1 showed higher peaks in ICU patients when compared to healthy controls. Bmal1 and CLOCK expression was blunted in septic patients.

The relationship between circadian rhythm disruption and clinical and environmental factors in critical illness has been largely unexplored. Maas *et al.* published two additional papers (using the original larger dataset of *n*=112 critically ill patients [33]) to associate changes in clock genes with melatonin levels, light/dark phase, nutritional intake, and physical activity levels [37^▪▪^,^38^^▪▪^]. No associations were found between clock gene amplitudes and illness severity (SOFA scores), encephalopathy (Glasgow Coma Score), rest-activity rhythmicity (daily pattern of activity and rest), and melatonin levels. Low day-time light intensity levels, frequent nursing care, and night-time noise were highly prevalent. Nutritional intake (only available in *n* = 43/112 patients) was inadequate, with 39% receiving some bolus feeding (enteral or oral); however, no detailed nutritional intake data was reported. Physical activity levels in critically ill patients were drastically lower compared with ambulatory and bedrest healthy controls. No relation between feeding regime and/or physical activity levels and clock gene expression was made, so it remains yet uncertain how disruptors such as light, noise, nutrition, and/or physical acitivity affects circadian rhythm in ICU patients.

## INTERMITTENT FEEDING AND METABOLIC OUTCOMES IN CRITICALLY ILL PATIENTS

The optimal feeding mode for critically ill patients has become an ongoing debate in critical care nutrition. As reviewed previously, most continuous versus intermittent feeding [12–17] studies in critical illness are aimed to improve nutritional intake targets. To date, the few studies that have been conducted have included relatively small patient cohorts and have failed to show clear clinical benefit; well-controlled RCTs comparing metabolic effects of altered meal timing in critically ill patients remain scarce. Supporting evidence to understand the possible effect of intermittent feeding on glucose, gastrointestinal, and muscle metabolism in critically ill patients is discussed in the following sections.

### Glycaemic control and gastrointestinal function

In critically ill patients, intermittent feeding has been shown to either increase glycaemic variability [39] or not to affect daily blood glucose levels [40,41], whereas reduced insulin requirements have been observed following intermittent feeding [39,42,43]. Gastrointestinal intolerance (e.g., delayed gastric emptying) is common in critically ill patients, resulting in impaired nutrient absorption and an increased risk for aspiration; intermittent feeding may increase gut motility and the release of postprandial gastrointestinal hormones. Studies to date have only assessed surrogate measures of gastrointestinal dysfunction and have been inconclusive, reporting no difference [39] or higher [44,45,46^▪^] gastric volumes following intermittent feeding. With glucoregulatory and appetite hormones playing an essential role in glycaemic control and gastric emptying, further studies assessing the effect of meal timing on glycaemic control and gastrointestinal function in critically ill patients are needed.

### Muscle metabolism

Intermittent feeding has been suggested to stimulate muscle protein synthesis to a greater extent than continuous feeding due to increased plasma amino acid availability and, as such, may serve as an effective strategy to attenuate muscle wasting in patients. The largest study (*n* = 127 patients) conducted with a primary interest in muscle by McNelly *et al.*[39] observed no difference in change of muscle cross-sectional area (mean change: −1.1%) over ten days of intermittent feeding when compared with standard continuous feeding. A secondary analysis of this study [47^▪▪^], demonstrated an attenuated urea-to-creatinine ratio trajectory (as a marker of muscle wasting) in intermittent feeding compared with continuously fed patients, suggesting that intermittent feeding might be preventing catabolism. However, in this multicentre RCT, the primary outcome was available in only *n* = 63/127 patients (by day 10), and several confounding factors, including higher protein and energy intakes in the intermittent feeding group as well as the methodology used to assess muscle mass, may explain the lack of observed benefit. In a smaller patient cohort of 59 ICU patients [40] (*only abstract available*), no difference in change of thickness and cross-sectional area of *rectus femoris* during seven days of intermittent versus continuous feeding was observed; however, the limited information available from this study makes it hard to evaluate. No research to date has assessed the effect of intermittent feeding versus continuous feeding on muscle protein synthesis rates in critically ill patients, which requires further investigation to understand the impact of altered meal timing on muscle metabolism.

## MEAL TIMING AND CIRCADIAN RHYTHMS: IS THERE AN EXPECTED EFFECT FOR THE INTENSIVE CARE UNIT?

Environmental entrainers such as sleep/wake phase, food intake, and physical activity can reset or re-align circadian clocks in peripheral tissues [2^▪^]. Eating during the inactive phase in animals completely inverts the expression of core clock genes in muscle, adipose tissue, and liver [48]. To date, this has been poorly investigated in humans. Only two studies have recently investigated the effects of limiting meal timing on circadian clocks through repeated tissue sampling in health. Lundell *et al.*[49^▪▪^] showed that time-restricted eating did not alter clock genes in muscle, but changes in the circadian regulation of metabolites, including amino acids, were observed. Zhao *et al.*[27^▪^] took four repeated adipose tissue biopsies over 24 h and observed that time-restricted eating restored 3 out of 12 clock genes and rhythm to 450 genes in adipose tissue that were arrhythmic at baseline (*personal communication, manuscript accepted, in preprint*). Two other studies have reported that time-restricted eating induced changes in clock genes at different time points: time-restricted eating between 8 am and 2 pm decreased PER1 at 8 pm and increased CRY1/2 and RORα at 8 am and 8 pm [50]. Increased amplitude in BMAL1, CRY1, PER2, and RORα was also reported in white blood cells of patients with type 2 diabetes who ate three meals in 12 h versus six meals in 15 h [51].

**FIGURE 1:**
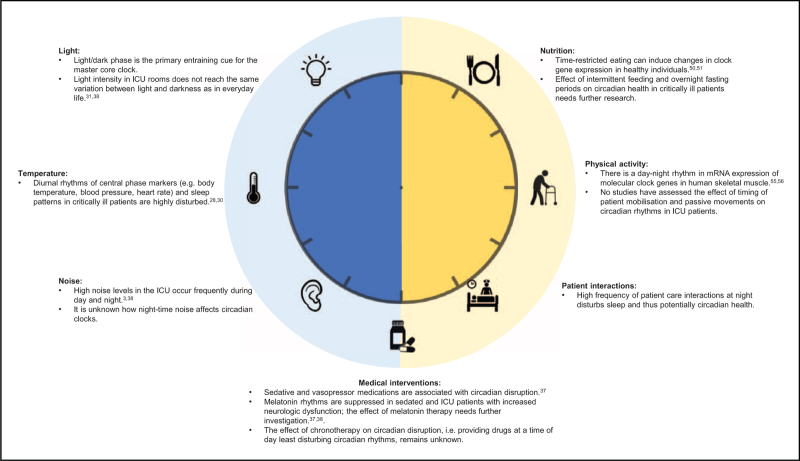
Clinical and environmental factors in the ICU that impact circadian rhythms.

No studies have been conducted to assess the impact of time of nutritional intake on circadian rhythms in critical illness. However, the time of day of meal ingestion affects the postprandial glucose response and shifting meal intake to earlier in the day improves glucose tolerance throughout the day in healthy adults and those with overweight or obesity [52,53]. In contrast to continuous feeding, intermittent feeding might reduce glucose intolerance and insulin resistance, which is relevant in critically ill patients, as up to 75% of patients show stress-induced hyperglycaemia [54]. The current clinical management of elevated glucose concentrations in the ICU is exogenous insulin administration with continuous enteral nutrition. However, intensive exogenous insulin therapy has been associated with negative consequences, including hypoglycaemic events, increased insulin administration, and increased mortality [55]. Moreover, continuous enteral nutrition to manage glucose levels is supported by limited evidence. Insulin (and IGF-1) has recently been recognised as a circadian entrainer and, as such, can serve as a primary signal of feeding time to cellular clocks throughout the body [19]. Therefore, intermittent feeding might be an effective strategy for preserving or re-aligning circadian rhythms, besides optimising glycaemic control. Moreover, incorporating overnight fasting periods has been suggested to be effective for metabolism, as research in healthy individuals has shown that fasting periods activate ketogenesis and autophagy [50]. A recent pilot study [56^▪▪^] tested the feasibility of a period of fasting (alternating 12 h feeding with 12 h fasting) in 70 prolonged critically ill patients, showing that a 12 h nutrient interruption can initiate a metabolic fasting response by increased serum bilirubin and plasma beta-hydroxybutyrate and decreasing insulin requirements and serum IGF-I. Although the effect of intermittent or cyclic feeding on metabolism and clinical outcomes in critically ill patients needs further investigation, intermittent feeding regimes including overnight fasting periods might be relevant to preserve or even reset circadian misalignment. Other potential modifiable clinical and environmental disruptors for circadian rhythms in ICU patients, including light, temperature, physical activity, noise, sleeping medication, and nursing and medical interventions, are summarised in Fig. 1[57^▪^,^58^].

## CONCLUSION

Studies in the last 18 months have further shown that intermittent feeding can increase nutritional intake. The suggested effect on improved glycaemic control, impaired gastric intolerance, and muscle mass maintenance compared to continuous feeding is minimal and based upon low-quality evidence. Ramifications on circadian misalignment in gastrointestinal, glucose, and muscle metabolism highlight the degree to which different tissues are affected, with studies in health showing that time-restricted eating can induce changes in peripheral clock genes. Chronotherapy, i.e., aligning meal timing, physical activity, and/or medication, are potential strategies to preserve or reset peripheral circadian rhythms; however, it is not known how these strategies can affect circadian rhythms in ICU patients. Interventional strategies to preserve circadian health could include the use of eye masks and earplugs, intermittent or cyclic day-time feeding, daily mobilisation, light-, and/or melatonin therapy during and post-ICU admission. However, the effect on metabolism and clinical outcomes is as yet unknown.

## Acknowledgements

*None.*

### Financial support and sponsorship

*I.W.K.K. holds a ZonMW/NWO Rubicon Fellowship and has received funding from the Diabetes Australia Research Program, American Society for Parenteral and Enteral Nutrition Rhoads Research Foundation, and the Royal Adelaide Hospital in relation to this work. L.K.H. has received funding from the National Health and Medical Research Council (Ideas Grant, #2012569), Medical Research Future Fund (Preventive and Public Health Grant, MRF1200555). A.R.H.V.Z. received honoraria for advisory board meetings, lectures, research, and travel expenses from Baxter, Braun, Cardinal Health, Danone-Nutricia, Dim-3, Fresenius Kabi, Mermaid, Lyric, and Nestle-Novartis.*

### Conflicts of interest

*There are no conflicts of interest.*

*Author contribution: I.W.K.K., L.K.H., and A.R.H.V.Z. were responsible for conceptualisation, data curation, and original draft writing and review and editing of the final manuscript.*

## REFERENCES AND RECOMMENDED READING

Papers of particular interest, published within the annual period of review, have been highlighted as:

▪ of special interest
▪▪ of outstanding interest

## References

[1] Rousseau AF Prescott HC Brett SJ. Long-term outcomes after critical illness: recent insights. Crit Care 2021; 25:108. 33731201 10.1186/s13054-021-03535-3 PMC7968190

[2▪] Regmi P Heilbronn LK. Time-restricted eating: benefits, mechanisms, and challenges in translation. iScience 2020; 23:101161. 32480126 10.1016/j.isci.2020.101161 PMC7262456

[3▪] Daou M Telias I Younes M. Abnormal sleep, circadian rhythm disruption, and delirium in the ICU: are they related? Front Neurol 2020; 11:549908. 33071941 10.3389/fneur.2020.549908 PMC7530631

[4] Kreymann KG Berger MM Deutz NE. ESPEN guidelines on enteral nutrition: intensive care. Clin Nutr 2006; 25:210–223. 16697087 10.1016/j.clnu.2006.01.021

[5] McClave SA Taylor BE Martindale RG. Guidelines for the provision and assessment of nutrition support therapy in the adult critically ill patient: Society of Critical Care Medicine (SCCM) and American Society for Parenteral and Enteral Nutrition (A.S.P.E.N.). J Parenter Enteral Nutr 2016; 40:159–211. 10.1177/0148607115621863 26773077

[6▪] Parr EB Heilbronn LK Hawley JA. A time to eat and a time to exercise. Exerc Sport Sci Rev 2020; 48:4–10. 31688298 10.1249/JES.0000000000000207 PMC6948807

[7] Bohe J Low JF Wolfe RR. Latency and duration of stimulation of human muscle protein synthesis during continuous infusion of amino acids. J Physiol 2001; 532 (Pt 2):575–579. 11306673 10.1111/j.1469-7793.2001.0575f.x PMC2278544

[8] Gazzaneo MC Suryawan A Orellana RA. Intermittent bolus feeding has a greater stimulatory effect on protein synthesis in skeletal muscle than continuous feeding in neonatal pigs. J Nutr 2011; 141:2152–2158. 22013195 10.3945/jn.111.147520 PMC3223872

[9] Evans DC Forbes R Jones C. Continuous versus bolus tube feeds: Does the modality affect glycemic variability, tube feeding volume, caloric intake, or insulin utilization? Int J Crit Illn Inj Sci 2016; 6:9–15. 27051616 10.4103/2229-5151.177357 PMC4795366

[10] Chowdhury AH Murray K Hoad CL. Effects of bolus and continuous nasogastric feeding on gastric emptying, small bowel water content, superior mesenteric artery blood flow, and plasma hormone concentrations in healthy adults: a randomized crossover study. Ann Surg 2016; 263:450–457. 25549202 10.1097/SLA.0000000000001110 PMC4741393

[11] Dirks ML Smeets JSJ Holwerda AM. Dietary feeding pattern does not modulate the loss of muscle mass or the decline in metabolic health during short-term bed rest. Am J Physiol Endocrinol Metab 2019; 316:E536–E545. 30645176 10.1152/ajpendo.00378.2018

[12] Pletschette Z Preiser JC. Continuous versus intermittent feeding of the critically ill: have we made progress? Curr Opin Crit Care 2020; 26:341–345. 32487846 10.1097/MCC.0000000000000733

[13] Puthucheary Z Gunst J. Are periods of feeding and fasting protective during critical illness? Curr Opin Clin Nutr Metab Care 2021; 24:183–188. 33538425 10.1097/MCO.0000000000000718

[14] Di Girolamo FG Situlin R Fiotti N. Intermittent vs. continuous enteral feeding to prevent catabolism in acutely ill adult and pediatric patients. Curr Opin Clin Nutr Metab Care 2017; 20:390–395. 28650855 10.1097/MCO.0000000000000397

[15] Bear DE Hart N Puthucheary Z. Continuous or intermittent feeding: pros and cons. Curr Opin Crit Care 2018; 24:256–261. 29877877 10.1097/MCC.0000000000000513

[16] Van Dyck L Casaer MP. Intermittent or continuous feeding: any difference during the first week? Curr Opin Crit Care 2019; 25:356–362. 31107308 10.1097/MCC.0000000000000617

[17] Patel JJ Rosenthal MD Heyland DK. Intermittent versus continuous feeding in critically ill adults. Curr Opin Clin Nutr Metab Care 2018; 21:116–120. 29232262 10.1097/MCO.0000000000000447

[18] Zheng X Sehgal A. AKT and TOR signaling set the pace of the circadian pacemaker. Curr Biol 2010; 20:1203–1208. 20619819 10.1016/j.cub.2010.05.027 PMC3165196

[19] Crosby P Hamnett R Putker M. Insulin/IGF-1 drives PERIOD synthesis to entrain circadian rhythms with feeding time. Cell 2019; 177:896–909 e20. 31030999 10.1016/j.cell.2019.02.017 PMC6506277

[20▪▪] Tuvia N Pivovarova-Ramich O Murahovschi V. Insulin directly regulates the circadian clock in adipose tissue. Diabetes 2021; 70:1985–1999. 34226282 10.2337/db20-0910

[21] Lamia KA Sachdeva UM DiTacchio L. AMPK regulates the circadian clock by cryptochrome phosphorylation and degradation. Science 2009; 326:437–440. 19833968 10.1126/science.1172156 PMC2819106

[22] Ramsey KM Yoshino J Brace CS. Circadian clock feedback cycle through NAMPT-mediated NAD+ biosynthesis. Science 2009; 324:651–654. 19299583 10.1126/science.1171641 PMC2738420

[23] Nakahata Y Sahar S Astarita G. Circadian control of the NAD+ salvage pathway by CLOCK-SIRT1. Science 2009; 324:654–657. 19286518 10.1126/science.1170803 PMC6501775

[24▪▪] Zeb F Wu X Chen L. Effect of time-restricted feeding on metabolic risk and circadian rhythm associated with gut microbiome in healthy males. Br J Nutr 2020; 123:1216–1226. 31902372 10.1017/S0007114519003428

[25▪▪] Mindikoglu AL Abdulsada MM Jain A. Intermittent fasting from dawn to sunset for 30 consecutive days is associated with anticancer proteomic signature and upregulates key regulatory proteins of glucose and lipid metabolism, circadian clock, DNA repair, cytoskeleton remodeling, immune system and cognitive function in healthy subjects. J Proteomics 2020; 217:103645. 31927066 10.1016/j.jprot.2020.103645 PMC7429999

[26] Mason IC Qian J Adler GK. Impact of circadian disruption on glucose metabolism: implications for type 2 diabetes. Diabetologia 2020; 63:462–472. 31915891 10.1007/s00125-019-05059-6 PMC7002226

[27▪] Zhao L Hutchison AT Wittert GA. Intermittent fasting does not uniformly impact genes involved in circadian regulation in women with obesity. Obesity 2020; 28 Suppl 1:S63–S67. 32438531 10.1002/oby.22775

[28] Acuna-Fernandez C Marin JS Diaz-Casado ME. Daily changes in the expression of clock genes in sepsis and their relation with sepsis outcome and urinary excretion of 6-sulfatoximelatonin. Shock 2020; 53:550–559. 31403491 10.1097/SHK.0000000000001433

[29] Diaz E Diaz I Del Busto C. Clock genes disruption in the intensive care unit. J Intensive Care Med 2019; 885066619876572. 10.1177/0885066619876572 31510864

[30] Davidson S Villarroel M Harford M. Day-to-day progression of vital-sign circadian rhythms in the intensive care unit. Crit Care 2021; 25:156. 33888129 10.1186/s13054-021-03574-w PMC8063456

[31▪▪] Okutan B Kjer CKW Poulsen LM. Sleep-wake rhythms determined by actigraphy during in-hospital stay following discharge from an intensive care unit. Acta Anaesthesiol Scand 2021; 65:801–808. 33590887 10.1111/aas.13800

[32] Yang PL Ward TM Burr RL. Sleep and circadian rhythms in survivors of acute respiratory failure. Front Neurol 2020; 11:94. 32117040 10.3389/fneur.2020.00094 PMC7033606

[33] 2020; Maas MB Iwanaszko M Lizza BD. Circadian gene expression rhythms during critical illness. crit care med. 48:e1294–e1299. 10.1097/CCM.0000000000004697 PMC7708445 33031153

[34] Diaz E Diaz I Del Busto C. Clock genes disruption in the intensive care unit. J Intensive Care Med 2020; 35:1497–1504. 31510864 10.1177/0885066619876572

[35] Coiffard B Diallo AB Culver A. Circadian rhythm disruption and sepsis in severe trauma patients. Shock 2019; 52:29–36. 30074979 10.1097/SHK.0000000000001241

[36] Hughes ME Abruzzi KC Allada R. Guidelines for genome-scale analysis of biological rhythms. J Biol Rhythms 2017; 32:380–393. 29098954 10.1177/0748730417728663 PMC5692188

[37▪▪] Maas MB Lizza BD Kim M. Stress-induced behavioral quiescence and abnormal rest-activity rhythms during critical illness. Crit Care Med 2020; 48:862–871. 32317592 10.1097/CCM.0000000000004334 PMC7242144

[38▪▪] Maas MB Lizza BD Abbott SM. Factors disrupting melatonin secretion rhythms during critical illness. Crit Care Med 2020; 48:854–861. 32317599 10.1097/CCM.0000000000004333 PMC7242161

[39] McNelly AS Bear DE Connolly BA. Effect of intermittent or continuous feed on muscle wasting in critical illness: A phase II clinical trial. Chest 2020. 10.1016/j.chest.2020.03.045 32247714

[40] Dong J Liu R Li L. [Effects of intermittent feeding and continuous feeding on muscle atrophy and nutritional status in critically ill patients]. Zhonghua Wei Zhong Bing Ji Jiu Yi Xue 2021; 33:844–848. 34412755 10.3760/cma.j.cn121430-20210408-00517

[41] Ren CJ Yao B Tuo M. Comparison of sequential feeding and continuous feeding on the blood glucose of critically ill patients: a noninferiority randomized controlled trial. Chin Med J (Engl) 2021; 134:1695–1700. 34397596 10.1097/CM9.0000000000001684 PMC8318659

[42] Sjulin TJ Strilka RJ Huprikar NA. Intermittent gastric feeds lower insulin requirements without worsening dysglycemia: a pilot randomized crossover trial. Int J Crit Illn Inj Sci 2020; 10:200–205. 33850829 10.4103/IJCIIS.IJCIIS_112_19 PMC8033209

[43] Seyyedi J Rooddehghan Z Mohammadi M. Comparison of the effect of enteral feeding through the bolus and continuous methods on serum phosphorus and glucose levels in patients with mechanical ventilation: a randomized clinical trial. J Nutr Metab 2020; 2020:6428418. 33005454 10.1155/2020/6428418 PMC7508222

[44] Zhu W Jiang Y Li J. Intermittent versus continuous tube feeding in patients with hemorrhagic stroke: a randomized controlled clinical trial. Eur J Clin Nutr 2020; 74:1420–1427. 32152512 10.1038/s41430-020-0579-6

[45] Satinsky I Richtarova J. Intermittent feeding in intensive care – the theory and practice. Rozhl Chir 2021; 100:66–73. 33910339

[46▪] Ma Y Cheng J Liu L. Intermittent versus continuous enteral nutrition on feeding intolerance in critically ill adults: a meta-analysis of randomized controlled trials. Int J Nurs Stud 2021; 113:103783. 33161333 10.1016/j.ijnurstu.2020.103783

[47▪▪] Flower L Haines RW McNelly A. Effect of intermittent or continuous feeding and amino acid concentration on urea-to-creatinine ratio in critical illness. J Parenter Enteral Nutr 2021. 10.1002/jpen.2258 34462921

[48] Abe T Kazama R Okauchi H. Food deprivation during active phase induces skeletal muscle atrophy via IGF-1 reduction in mice. Arch Biochem Biophys 2019; 677:108160. 31639326 10.1016/j.abb.2019.108160

[49▪▪] Lundell LS Parr EB Devlin BL. Time-restricted feeding alters lipid and amino acid metabolite rhythmicity without perturbing clock gene expression. Nat Commun 2020; 11:4643. 32938935 10.1038/s41467-020-18412-w PMC7495469

[50] Jamshed H Beyl RA Della Manna DL. Early time-restricted feeding improves 24-h glucose levels and affects markers of the circadian clock, aging, and autophagy in humans. Nutrients 2019; 11:1234. 10.3390/nu11061234 PMC6627766 31151228

[51] Jakubowicz D Landau Z Tsameret S. Reduction in glycated hemoglobin and daily insulin dose alongside circadian clock upregulation in patients with type 2 diabetes consuming a three-meal diet: a randomized clinical trial. Diabetes Care 2019. 10.2337/dc19-1142 31548244

[52] Davis R Bonham MP Nguo K. Glycaemic response at night is improved after eating a high protein meal compared with a standard meal: a cross-over study. Clin Nutr 2020; 39:1510–1516. 31303526 10.1016/j.clnu.2019.06.014

[53] Parr EB Devlin BL Radford BE. A delayed morning and earlier evening time-restricted feeding protocol for improving glycemic control and dietary adherence in men with overweight/obesity: a randomized controlled trial. Nutrients 2020; 12:505. 10.3390/nu12020505 PMC7071240 32079327

[54] Plummer MP Bellomo R Cousins CE. Dysglycaemia in the critically ill and the interaction of chronic and acute glycaemia with mortality. Intensive Care Med 2014; 40:973–980. 24760120 10.1007/s00134-014-3287-7

[55] Investigators N-SS Finfer S Chittock DR. Intensive versus conventional glucose control in critically ill patients. N Engl J Med 2009; 360:1283–1297. 19318384 10.1056/NEJMoa0810625

[56▪▪] Van Dyck L Vanhorebeek I Wilmer A. Towards a fasting-mimicking diet for critically ill patients: the pilot randomized crossover ICU-FM-1 study. Crit Care 2020; 24:249. 32448392 10.1186/s13054-020-02987-3 PMC7245817

[57▪] Held NM Wefers J van Weeghel M. Skeletal muscle in healthy humans exhibits a day-night rhythm in lipid metabolism. Mol Metab 2020; 37:100989. 32272236 10.1016/j.molmet.2020.100989 PMC7217992

[58] van Moorsel D Hansen J Havekes B. Demonstration of a day-night rhythm in human skeletal muscle oxidative capacity. Mol Metab 2016; 5:635–645. 27656401 10.1016/j.molmet.2016.06.012 PMC5021670

